# Neuropsin Inactivation Has Protective Effects against Depressive-Like Behaviours and Memory Impairment Induced by Chronic Stress

**DOI:** 10.1371/journal.pgen.1006356

**Published:** 2016-10-04

**Authors:** Simon Chang, Philane Bok, Cheng-Pu Sun, Andrew Edwards, Guo-Jen Huang

**Affiliations:** 1 Department and Graduate Institute of Biomedical Sciences, College of Medicine, Chang Gung University, Tao-Yuan, Taiwan; 2 Institute of Biomedical Sciences, Academia Sinica, Taipei, Taiwan; 3 Ninewells Hospital and Medical School, University of Dundee, Dundee, United Kingdom; 4 Neuroscience Research Center, Chang Gung Memorial Hospital, Linkou Medical Center, Taoyuan, Taiwan; University of Texas Health Science Center at San Antonio, UNITED STATES

## Abstract

Mounting evidence suggests the interaction between stress and genetics contribute to the development of depressive symptoms. Currently, the molecular mechanisms mediating this process are poorly understood, hindering the development of new clinical interventions. Here, we investigate the interaction between neuropsin, a serine protease, and chronic stress on the development of depressive-like behaviours in mice. We found no difference in baseline behaviour between neuropsin knockout and wild-type mice. However, our results show that neuropsin knockout mice are protected against the development of depressive-like behaviours and memory impairment following chronic stress. We hypothesised that this difference in behaviour may be due to an interaction between neuropsin and elevated plasma corticosterone. To test this, we subjected mice to chronic corticosterone injections. These injections resulted in changes to hippocampal structure similar to that observed following chronic stress. We found that inactivation of neuropsin limits the extent of these anatomical changes in both the chronic stress and the corticosterone injection exposed cohorts. We next used viral vectors to knockdown or overexpress neuropsin in the hippocampus to confirm the results of the KO study. Additionally, we found that inactivation of neuropsin limited glutamate dysregulation, associated with increased generation of reactive oxygen species, resulting from prolonged elevated plasma corticosterone. In this study, we demonstrate that neuropsin inactivation protects against the impairment of hippocampal functions and the depressive-like behaviour induced by chronic stress or high levels of corticosterone. Consequently, we suggest neuropsin is a potential target for clinical interventions for the management of stress disorders.

## Author Summary

Depression is a medical condition that results in significant morbidity, mortality and reduced quality of life. Understanding the molecular mechanism in which stress leads to depression is essential for the discovery of new clinical interventions. Currently, despite considerable research, the mechanisms underlying stress-related illnesses are unclear. In this study, we reveal a novel link between the interaction of serine protease neuropsin with corticosterone and the development of chronic stress induced depressive-like behaviour. We found no difference in baseline behaviour between neuropsin knockout and wild-type mice. However, our results show that neuropsin knockout mice are protected against the development of depressive-like behaviours and memory impairment following chronic stress. We suggest the dysregulation of the glutamate system may lead to increased reactive oxygen species and act as a possible mechanism in which stress changes hippocampal architecture. We found that inactivation of neuropsin limits the extent of these anatomical changes in both chronic stress and corticosterone injection exposed cohorts. In this study, we outline a mechanism that may open new possibilities for the treatment of stress-related psychiatric disorders.

## Introduction

Stress, especially chronic stress, can lead to disorders such as depression and consequently result in significant morbidity and reduced quality of life [1]. Despite considerable research, the mechanisms underlying stress-related illnesses are unclear. Neuropsin (NP, also named *Klk8*) is a serine protease that exhibits trypsin-like activity with a strong preference for Arg in the P1 position [2]. Its substrates include neuregulin-1, fibronectin, vitronectin, synaptic adhesion molecule L1, and Ephrin type-B receptor 2 (EphB2) [3–6]. Neuropsin is predominantly expressed in the amygdala and CA regions of the hippocampus [6–8]. These two regions have been long associated with stress, emotion, learning and memory processes [9–11]. There are several pieces of evidence suggesting a link between neuropsin and stress. First, stress increases the expression of neuropsin mRNA in the hippocampus through a glucocorticoid-sensitive pathway [12]. Second, neuropsin has been shown to affect stress-induced anxiety by cleaving the extracellular portion of EphB2 [6]. Finally, the antidepressant fluoxetine alters the expression of neuropsin in the hippocampus [13]. However, the importance of neuropsin in chronic stress induced impairment of brain function and behaviour still remains unknown.

In this study, we sought to investigate the role of neuropsin in the regulation of depressive-like behaviour, learning and memory following chronic stress. It is well known that stress induces a rapid rise in corticosterone. Chronically elevated corticosterone impairs memory, reduces neuronal spine density, and decreases hippocampal neurogenesis and volume [14–17]. Therefore, we further explored the interaction between corticosterone and neuropsin and the associated effects on behaviour, gene expression and hippocampal architecture. We investigated the role of neuropsin using multiple models: a knock out; a lenti-viral vector induced knock down; and an AAV vector induced overexpression model. Our results suggest that understanding the role of neuropsin in the brain may open new possibilities for the treatment of stress-associated disorders.

## Results

### The severity of depressive-like behaviours induced by chronic stress is reduced in neuropsin-deficient mice

To investigate whether neuropsin expression is influenced by stress, we examined the expression of neuropsin in the hippocampus following acute and chronic restraint exposure. Eight week old C57BL/6 male mice were subjected to restraint stress for 30 min to model acute stress (n = 4) or for 28 days to model chronic stress (n = 8). Mice were killed and hippocampal neuropsin mRNA was quantified with real-time PCR. The data show hippocampal neuropsin expression was increased after both acute restraint stress (mean ± SEM; control: 1.00 ± 0.09; stress: 1.5 ± 0.17, *p* = 0.036, *t* = 2.68) and 28 days chronic stress (control: 1.00 ± 0.192; stress: 1.94 ± 0.24, *p* = 0.029, *t* = 2.53).

**Fig 1:**
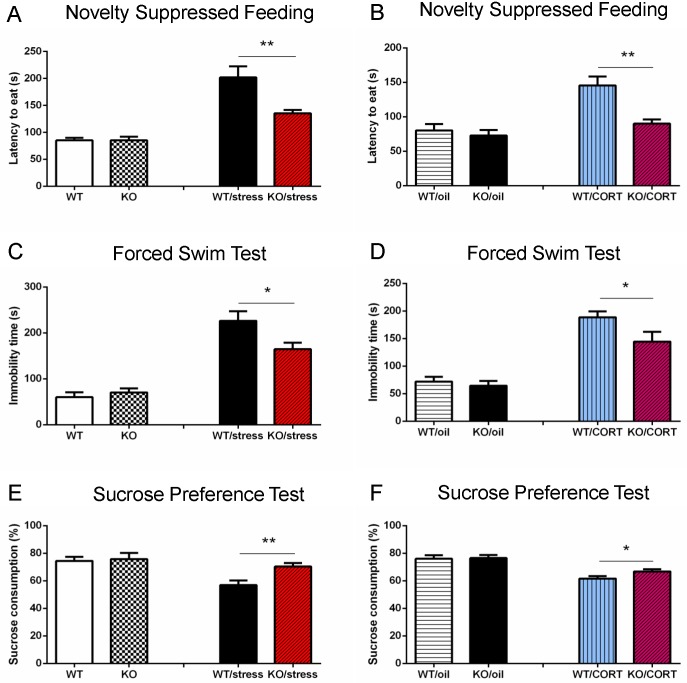
Neuropsin-deficient mice are resistant to chronic stress or elevated corticosterone induced depressive-like behaviour. (A, C, E) There is no significant difference between WT and KO mice in the novelty suppressed feeding, forced swim or sucrose preference tests without prior exposure to stress. After chronic stress, neuropsin KO mice approach food pellets more quickly than WT mice in the novelty suppressed feeding test. In the forced swim test, the immobility time of KO mice is shorter than WT mice. KO mice drink more sucrose than WT mice in the sucrose preference test. (B, D, F) There is no significant difference between WT and KO mice in the oil injection group. After chronic corticosterone injection (20 mg/kg/day), neuropsin KO mice show less depressive-like behaviour in novelty suppressed feeding, forced swim test, and sucrose preference test than WT mice. n = 8–10 in each group, values represent mean ± SEM. * *p* < 0.05, ** *p* < 0.01.

Next, we used neuropsin knockout (KO) mice (n = 8) and wild-type (WT) mice (n = 10) to conduct tests to assess depressive-like behaviour; this included novelty suppressed feeding, forced swim, and a sucrose preference test. Our data showed there was no difference between WT and KO mice for depressive-like behaviour (Fig 1A, 1C and 1E). Whilst a previous study suggested that neuropsin KO mice exhibit less anxiety compared to WT following acute restraint stress [6], the role of neurospin in chronic stress remained unclear. To study this, both KO (n = 9) and WT (n = 10) mice were subjected to a battery of restraint stress, footshock and sleep deprivation for 28 days (one stress paradigm per day). Behavioural tests were started on the 12th day of the 28 day stress protocol. We first measured the activity of mice using an open field test. The result showed there was an effect from stress but there was no significant difference between the WT and KO groups (effect of stress, *F*_1,34_ = 10.33, *p* = 0.003; NP, *p* = 0.28) (S1A Fig). In the novelty suppressed feeding test, KO mice approached food pellets quicker than WT mice (*p* = 0.001, *t* = 3.83). For the forced swim test, the immobility time of KO mice was less than WT (*p* = 0.031, *t* = 2.37). Additionally, neuropsin KO mice drank more sucrose in the sucrose preference test (*p* = 0.0054, *t* = 3.18) (Fig 1A, 1C and 1E). These results indicate that neuropsin KO mice showed less depressive-like behaviour following chronic stress compared to littermate WT mice.

### Mice lacking neuropsin feature a reduction in the impairment of both memory and hippocampal neurogenesis observed following chronic stress

**Fig 2:**
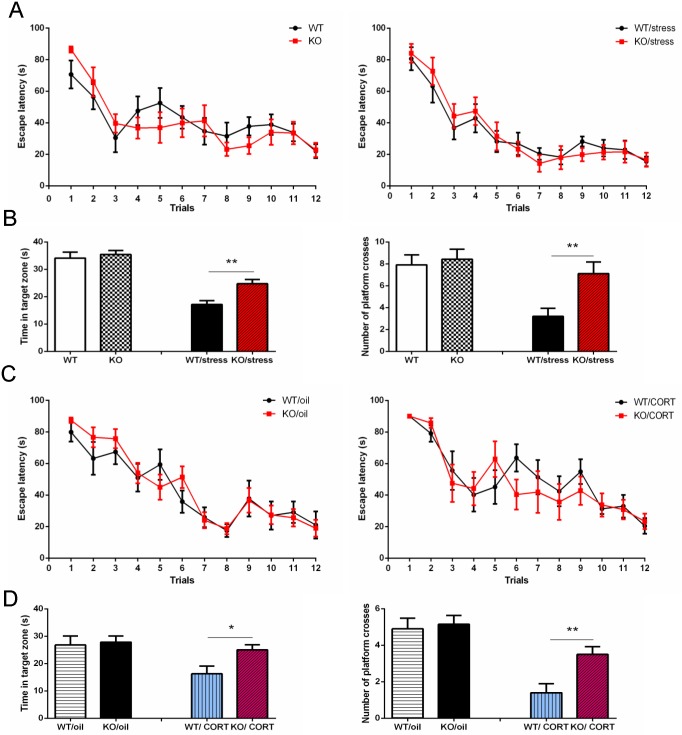
Inactivation of neuropsin reduces the impairment of spatial memory performance following chronic stress or elevated corticosterone. (A) There is no significant difference in the water maze training curve between KO and WT mice. In the chronic stress groups, there is also no difference in the training curve. (B) Two weeks later, there is no significant difference in memory as measured by time spent in the target zone and the number of phantom platform crosses; however in the chronic stress group, neuropsin KO mice spent more time in the target zone and made a greater number of phantom platform crosses. (C) In both oil and corticosterone injection groups (20 mg/kg/day) there is no difference in the training curves. (D) KO mice performed significantly better in memory tests that WT following corticosterone injection. n = 10–11 in each group, values represent mean ± SEM. * *p* < 0.05, ** *p* < 0.01.

Memory is affected by numerous factors including stress and depression. Neuropsin has been suggested to be essential for memory acquisition, although some inconsistencies exist in the literature [18–21]. To further investigate whether neuropsin is involved in learning and memory following chronic stress, we used two independent cohorts of mice to perform a water maze test. First, we measured the basal level of learning and memory. WT and KO mice (n = 11) had four trials a day for three days. Assessments of spatial memory were obtained by placing mice in the water maze without a platform two weeks after training and recording the time spent in the target zone and the number of times they crossed the phantom platform location. Results show there was no difference between the two training curves (escape latency) (Repeated measures ANOVA, *p =* 0.69, *F*_11,220_ = 0.16). In the memory test, there were also no differences in the time spent in the target zone (*p* = 0.33, *t* = 0.98) and the number of phantom platform crosses (*p* = 0.68, *t* = 0.41) (Fig 2A and 2B). These data indicate that neuropsin inactivation does not affect basal learning and memory as measured in a water maze. Next, we assessed spatial learning and memory in another cohort of mice (n = 10) which had experienced chronic stress (training started from day 12). Results showed no difference in the training curves (*p* = 0.81, *F*_11,187_ = 0.054). However, the memory test showed KO mice spent more time in the target zone (*p* = 0.0018, *t* = 3.69) and made more phantom platform crosses (*p* = 0.0069, *t* = 3.07) (Fig 2A and 2B). These results suggest that in mice exposed to chronic stress, a lack of neuropsin does not influence spatial learning, but does improve indicators of memory.

Neurogenesis has the effect of buffering stress and depressive-like behaviours and has long been associated with learning and memory [22, 23]. Previous research has demonstrated the status of the CA3 region can affect the survival of newly born neurons in the dentate gyrus [24]. Here, we asked whether neuropsin plays a role in the regulation of adult hippocampal neurogenesis following chronic stress. Mice were divided into four groups (WT, KO, n = 7; WT, KO with chronic stress, n = 10 in each group). The day prior to the commencement of the chronic stress protocol, mice were injected with 5-bromo-2'-deoxyuridine (BrdU) (200 mg/kg, i.p.) to label newly born cells. Mice were killed 28 days after BrdU injection to evaluate cell survival of new neurons generated at the time of BrdU injection. To assess proliferation of neuronal progenitors at the time of culling, we stained for doublecortin (DCX), a marker for immature neurons, and KI67, a marker for cell proliferation.

**Fig 3:**
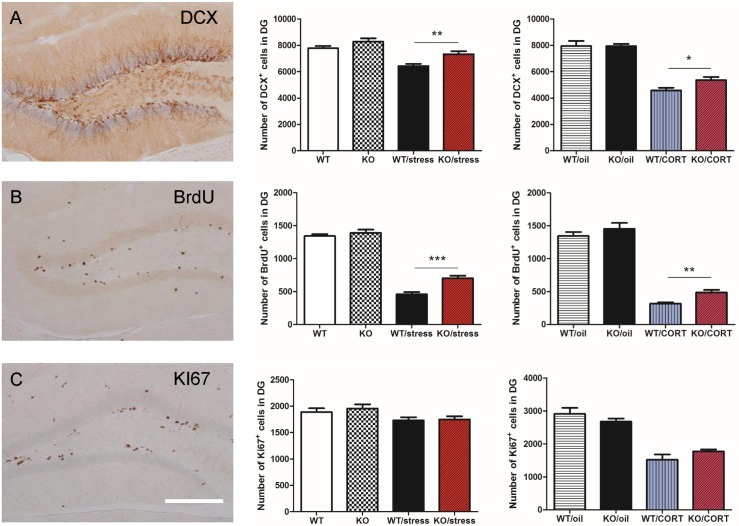
Neuropsin-deficient mice are partially protected against the effect on hippocampal neurogenesis elicited by chronic stress or corticosterone injection. (A) Chronic stress (n = 7–10) or chronic corticosterone injections (n = 6) dramatically decrease hippocampal neurogenesis. Neuropsin KO mice have a higher cell count of DCX positive cells in the dentate gyrus. (B) There are more BrdU positive cells in the dentate gyrus in KO mice than WT mice. (C) There is no significant difference in KI67 cell counts in both groups; n = 7–10 for chronic stress groups, n = 6 for chronic corticosterone groups. Scale bar = 200μm, values represent mean ± SEM. * *p* < 0.05, ** *p* < 0.01.

A two-way ANOVA revealed a significant stress effect on neurogenesis (DCX: *F*_1,29_ = 30.7, *p* < 0.0001; BrdU: *F*_1,29_ = 389.7, *p* < 0.0001, KI67: *F*_1,29_ = 7.6, *p =* 0.01). For the DCX, BrdU, and KI67 cell counts in the dentate gyrus, there were no significant differences between KO and WT mice (*p* > 0.05). In the chronic stress groups, we detected more DCX (*p* = 0.002, *t* = 3.3) and BrdU (*p* < 0.0001, *t* = 4.71) positive cells in the dentate gyrus of KO mice compared to WT mice. However, there was no significant difference in KI67 cell count (*p* = 0.86, *t* = 0.17) (Fig 3A–3C). Overall, these results suggest neuropsin does not affect neurogenesis under normal conditions but does play a role, especially in cell survival, when mice experience chronic stress.

### Neuropsin interacts with corticosterone to influence behaviour and hippocampal architecture

Corticosterone is released in response to stress. To confirm whether the stress models we used increased plasma corticosterone, we measured basal levels in both neuropsin WT and KO mice (n = 8) and levels after 3 hrs of restraint stress, 30 min after footshock, after 6 hrs of sleep deprivation and 24 hrs after all stressors were completed. Our result showed that the stressors we used in this study elevated plasma corticosterone (Two way ANOVA; *F*_4,56_ = 91.24, *p* < 0.0001) and there was no significant difference between WT and KO mice (basal, *p* = 0.69, *t* = 0.4; restraint, *p* = 0.15, *t* = 1.5; footshock, *p* = 0.77, *t* = 0.29; sleep deprivation, *p* = 0.16, *t* = 0.48; 24 hrs after stress, *p* = 0.34, *t* = 0.98) (S2 Fig).

In our experiment, we sought to establish whether our findings on behaviour after chronic stress were due to an interaction between elevated corticosterone and neuropsin inactivation. To do so, KO and WT mice were injected with oil (KO/oil, n = 12 and WT/oil, n = 10) or corticosterone (KO/CORT, n = 8 and WT/CORT, n = 10, 20 mg/kg/day, subcutaneously) daily for 32 days to imitate the elevated corticosterone during chronic stress. On day 12, we conducted a battery of tests for assessing depressive-like behaviour, learning and memory.

No differences were found in any of the behavioural tests from the oil injected groups. (Figs 1B, 1D, 1F and 2C and 2D). However, in the corticosterone injected groups, we found that KO mice exhibited less depressive-like behaviour than WT mice. Corticosterone significantly decreased activity but there was no significant difference between WT and KO groups (effect of corticosterone, *F*_1,37_ = 0.3, *p* < 0.0001; effect of neuropsin *P* = 0.58) (S1B Fig). KO mice had a reduced latency to approach food pellets in the novelty suppressed feeding test (*p* = 0.002, *t* = 3.54), reduced immobility time in the forced swim test (*p* = 0.039, *t* = 2.22) and drank more sucrose than WT mice in the sucrose preference test (*p* = 0.04, *t* = 2.18) (Fig 1B, 1D and 1F). On day 17, mice were trained in the water maze, and memory performance was assessed two weeks later. There was no difference in the training curves between WT and KO (*p* = 0.54, *F*_11,176_ = 0.39). However, for the memory test, the KO mice spent more time in the target zone (*p* = 0.026, *t* = 2.44) and made more phantom platform crosses (*p* = 0.006, *t* = 3.11) (Fig 2C and 2D). These results suggest that corticosterone interacts with neuropsin to affect spatial memory performance.

Evidence shows that hippocampal neurogenesis is inhibited by corticosterone [25]. Consequently, we sought to ascertain whether neuropsin interacts with corticosterone to regulate neurogenesis. Our data shows that corticosterone injection strongly reduced hippocampal neurogenesis (DCX: *F*_1,20_ = 131.6, *p* < 0.0001; BrdU: *F*_1,20_ = 71.96, *p* < 0.0001; KI67: *F*_1,20_ = 75.64, *p* < 0.0001). However, there was a significantly higher level of DCX (*p* = 0.043, *t* = 2.15) and BrdU (*p* = 0.003, *t* = 3.38) cell counts, but not KI67 (*p* = 0.19, t = 1.34) in the dentate gyrus in the neuropsin KO compared to WT (Fig 3A–3C). This suggests neuropsin interacts with plasma corticosterone to influence cell survival but not proliferation in the dentate gyrus.

**Fig 4:**
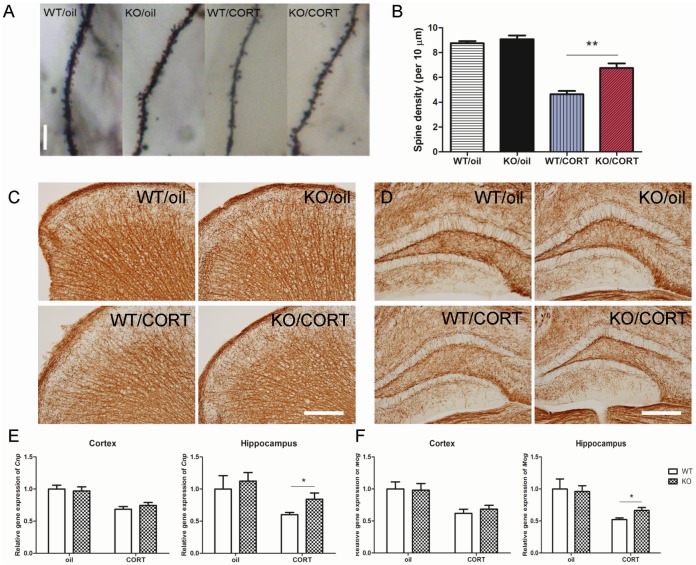
Neuropsin interacts with corticosterone to influence dendritic spine density and myelination. (A, B) The hippocampal dendritic spine density decreases in both WT and KO mice following chronic corticosterone injections but KO mice preserve more dendritic spines compared to WT mice, n = 4, Scale bar = 10μm. (C, D) Immunohistochemistry staining of CNPase in cortex and hippocampus. Scale bar = 200μm. (E, F) After chronic corticosterone injections, KO mice retain a higher expression of *Cnp* and *Mog* compared to WT mice in the hippocampus but there is no significant difference in the cortex. n = 6 in each group, values represent mean ± SEM. * *p* < 0.05, ** *p* < 0.01.

In regards to dendritic morphology, it is known that elevated corticosterone reduces hippocampal dendritic spine density [26], which has been suggested to be important for learning and memory [27]. To better understand whether neuropsin interacts with corticosterone to affect dendritic structure, we utilised Golgi staining to analyse the spine density in CA3. Our data showed corticosterone reduced spine density significantly (*F*_1,12_ = 127.3, *p* < 0.0001). There was no significant difference in spine density between WT and KO mice with oil injection (*p* = 0.42, *t* = 0.82). In the corticosterone injected groups, KO mice exhibited a higher spine density than WT mice (*p* = 0.0002, *t* = 5.22) (Fig 4A and 4B).

Stress may accelerate the ageing process and accelerate neurodegeneration [28]. Previous studies have shown that neuropsin is expressed in oligodendrocytes after injury to the central nervous system [29] and that prolonged corticosterone treatment of adult rats inhibits the proliferation of oligodendrocyte progenitors [30]. To better understand the role of neuropsin in the regulation of demyelination, we quantified *Cnp* (2',3'-Cyclic-nucleotide 3'-phosphodiesterase) and *Mog* (Myelin Oligodendrocyte Glycoprotein) expression from hippocampus and cortex derived mRNA after chronic oil or corticosterone injection (n = 6). There was no significant difference between WT and KO in both the hippocampus and cortex in the oil injected groups. However, in the corticosterone injected groups, whilst there was no difference in the cortex, there was more expression of *Cnp* (*p* = 0.04, *t* = 2.23) and *Mog* (*p* = 0.03, *t* = 2.4) in the hippocampus of KO mice (Fig 4C–4F).

Collectively, these data demonstrate that mice lacking neuropsin feature a protective effect on neurogenesis, dendritic morphology, and demyelination in the hippocampus after chronic elevated plasma corticosterone.

### Neuropsin inactivation decreases hippocampal neuronal activity following corticosterone injection

**Fig 5:**
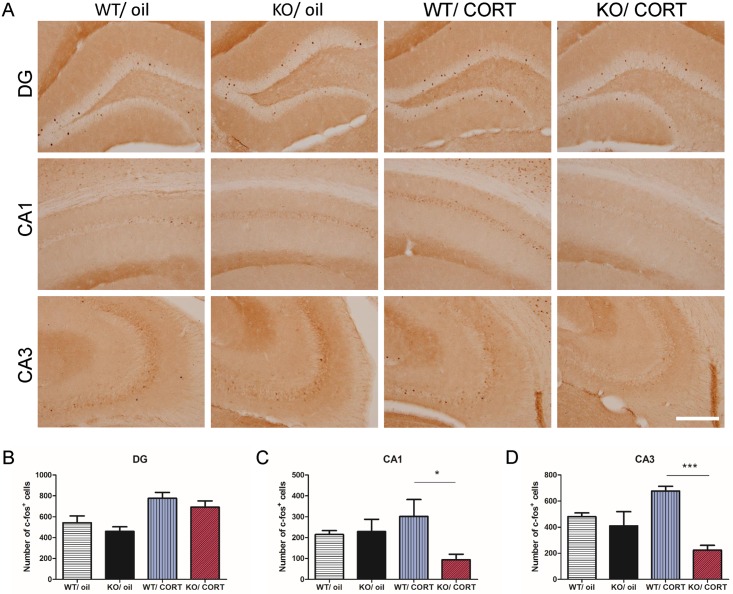
Inactivation of neuropsin blocks corticosterone induced hippocampal neuronal activity. (A) One day after chronic corticosterone or oil injection in WT and KO mice, c-Fos positive cells in the dentate gyrus (DG), CA1 and CA3. (B-D) Quantification of c-Fos positive cells in the DG, CA1, and CA3. KO/CORT mice display fewer c-Fos positive cells in the CA1 and CA3 compared to WT/CORT mice. n = 6 in each group, scale bar = 200 μm, values represent mean ± SEM. * *p* < 0.05, *** *p* < 0.001.

The expression of c-Fos is commonly used as a neuronal activity marker following stimulus [31]. In order to better understand the neuronal activity in WT and KO hippocampi in response to high levels of corticosterone, we measured neuronal activation by detecting the number of c-Fos positive cells twenty-four hours after the last injection of corticosterone or oil (28 days of injections) in both WT and KO mice (n = 6 in each group). In the oil treated groups, our results show there was no difference between WT and KO mice. Interestingly, after corticosterone injection, WT mice had more c-Fos positive cells in the dentate gyrus (DG) (*p* = 0.02, *t* = 2.7) and CA3 (*p* = 0.001, *t* = 4.3), but not in the CA1 (*p* = 0.31. *t* = 1.06). Meanwhile, KO/CORT mice featured significantly fewer c-Fos positive cells in CA1 (*p* = 0.03, *t* = 2.43) and CA3 (*p* < 0.0001, *t* = 8.87), but no significant difference in DG (*p* = 0.33, *t* = 1.01) compared to WT/CORT mice (Fig 5). These results demonstrate that KO mice exhibit a reduced response to corticosterone treatment as measured by hippocampal c-Fos expression.

### AAV mediated overexpression of neuropsin leads to depressive-like behaviour and memory impairment following corticosterone injection

One of the major limitations of using constitutive KO animals is that aberrant development may potentially influence data. The KO model may have an effect on somatic cells and brain regions other than the hippocampus. To address this, we overexpressed neuropsin in the hippocampi of KO mice via an AAV9-CB-neuropsin viral vector (NP/OE, n = 8) together with an AAV9-CB-GFP control (NP/KO, n = 8). Two weeks after surgery, we started to inject mice with corticosterone (20 mg/kg/day). After 14 days of corticosterone injections, we subjected the mice to a forced swim test, novelty suppressed feeding test and a sucrose preference test. Interestingly, NP/OE mice have longer immobility times in the forced swim test (*p* = 0.003, *t* = 3.62), and a greater latency to approach food pellets in the novelty suppressed feeding test (*p* = 0.018, *t* = 2.72) in comparison with NP/KO mice. For the sucrose preference test, there was no significant difference between the two groups (*p* = 0.09, *t* = 1.84) (S3A Fig). Our data shows that overexpression of neuropsin with concordant corticosterone injections leads to depressive-like behaviour.

Next, we performed a water maze test to measure spatial learning and memory. There was no significant difference between the learning curves of both groups (*p* = 0.99, *F*_11,66_ = 0.0004). We performed a memory test two weeks after training. The results showed no significant difference spent in the target zone between groups (*p* = 0.172, *t* = 1.45) but the number of times NP/OE mice crossed the phantom platform was significantly fewer (*p* = 0.03, *t* = 2.33). This suggests overexpression of neuropsin may further impair spatial memory after chronic corticosterone injection (S3B Fig). We confirmed hippocampal neuropsin overexpression by using qPCR. NP/OE mice featured a dramatic increase in neuropsin mRNA compared with NP/KO mice and NP/WT mice (one- way ANOVA, *p* = 0.0006, *F*_2,12_ = 14.96) (S4B Fig).

### Lentiviral vector knockdown of neuropsin attenuates depressive-like behaviour and memory impairment after corticosterone injection

In contrast to the overexpression group, we performed surgery on wild type C57BL/6 mice (n = 9) and injected either scramble sequence control or lentiviral vector shRNA to knockdown neuropsin in the hippocampus. Two weeks after surgery, we injected corticosterone daily on all mice. After 14 days of corticosterone injection, we evaluated the mice using the behaviour tests described above. Mice injected with the control scrambled viral vector featured a longer immobility time in the forced swim test (*p* < 0.0001, *t* = 6.34) and took a longer time to approach a food pellet in the novelty suppressed feeding test (*p* = 0.01, *t* = 2.74). There was no significant difference between control and knockdown in the sucrose preference test (*p* = 0.06, *t* = 2.04) (S3C Fig). Overall, these data suggest that inactivation of neuropsin protects against depressive-like behaviour.

Next, we performed a water maze test to evaluate spacial learning and memory. The results showed no significant difference in the learning curves between groups (*p* = 0.06, *F*_11,88_ = 4.49). Again, we performed the memory test two weeks after training. The results were consistent with the overexpression group: knockdown mice made significantly more platform crosses than the control group (*p* = 0.02, *t* = 2.39) but there was no significant difference in time spent within the target zone (*p* = 0.18, *t* = 1.38) (S3D Fig). The expression analysis showed shRNA lentiviral vector significantly decrease neuropsin expression compared to the scramble control (*p* < 0.0001, *t* = 7.73) (S4C Fig).

### Inactivated neuropsin prevents glutamate dysregulation and lowers reactive oxygen species production elicited by corticosterone

**Fig 6:**
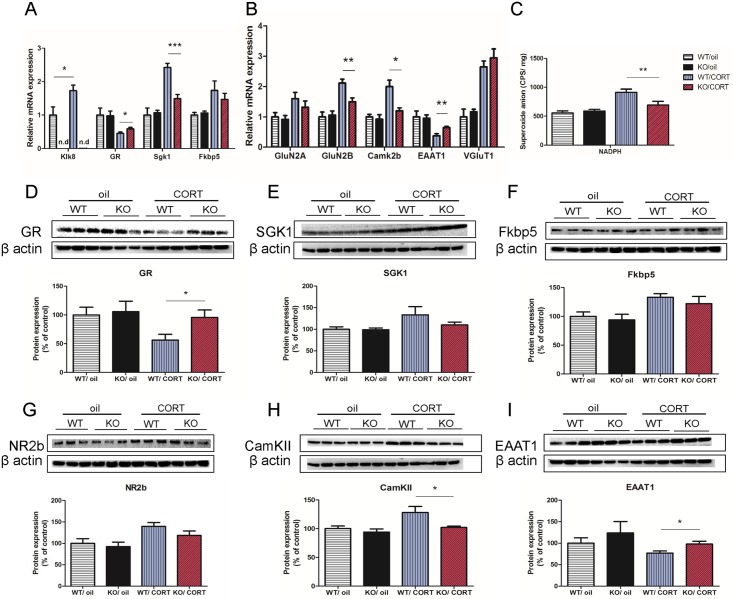
Neuropsin-deficient mice are resistant to glutamate transmission dysregulation induced by chronic elevated plasma corticosterone. (A) Gene expressions of *Klk8*, *GR*, *Sgk1* and *Fkbp5* in the hippocampus in WT and KO mice after chronic corticosterone or oil injection. (B) Gene expressions of glutamate receptors, *GluN2A*, *GluN2B* and *Camk2b* and glutamate reuptake (EAAT1), vesicle packing (VGluT1) in hippocampus. (C) Detection of NADPH induced reactive oxygen species in the hippocampus of WT and KO mice after chronic corticosterone or oil injection. n = 9–13 in each group. (D-I) Protein expressions of GR, SGK1, Fkbp5, NR2b, EAAT1 and CamKII measured by western blot assay. n = 6 in each group, values represent mean ± SEM. * *p* < 0.05, ** *p* < 0.01.

To further investigate the mechanism of how neuropsin interacts with corticosterone to affect animal behaviour and hippocampal architecture, we analysed hippocampal gene and protein expression in four groups (n = 6 in each group): WT/oil, KO/oil, WT/CORT, and KO/CORT. We first measured the hippocampal gene expression of neuropsin (*Klk8*). As expected, *Klk8* expression was not detectable in KO mice. However, *Klk8* expression was increased after chronic corticosterone injection in WT mice (*p* = 0.03, *t* = 2.46). For GR (glucocorticoid receptor), KO/CORT mice had higher expression compared to WT/CORT mice (*p* = 0.04, *t* = 2.3). This change can also be detected at the protein level (*p* = 0.03, *t* = 2.45) (Fig 6A and 6D). Next, we analysed GR downstream target genes: serum and glucocorticoid-regulated kinase 1 (*Sgk1*) and FK506 binding protein 5 (*Fkbp5*). *Sgk1* is thought to transfer N-methyl-D-aspartate (NMDA) receptors to the plasma membrane and have a regulating function in learning and memory and also mediate the effect of glucocorticoids on neurogenesis and brain function including learning and memory [32–34]. *Fkbp5* is highly associated with depression and other psychiatric disorders and is thought to reduce ligand sensitivity of the GR and be involved in stress response [35, 36]. KO/CORT mice showed a lower level of *Sgk1* expression compared to WT/CORT mice (*p* = 0.0004, *t* = 5.25) and no difference in *Fkbp5* (*p* = 0.25, *t* = 1.27) (Fig 6A, 6E and 6F). With regard to protein levels, western blot results show no differences in both SGK1 and Fkbp5 protein expression. These results provide evidence that mice lacking neuropsin have a reduced response to elevated plasma corticosterone.

It has been suggested that the imbalance of glutamate transmission is pathogenic in mood disorders [37]. Therefore, we measured the expression of regulatory genes that are involved in the removal (reuptake) of glutamate from the neuronal synaptic cleft in the hippocampus. We show that corticosterone injection reduces the expression of excitatory amino-acid transporter 1 (EAAT1) gene expression (F_1,20_ = 15.63, *p* = 0.0008). In corticosterone injected mice, KO mice have a higher level of EAAT1 gene expression (*p* = 0.008, *t* = 3.23) than WT controls. This difference between genotypes is also observed at the protein level (*p* = 0.027, *t* = 2.59) (Fig 6B and 6I). Expression of the vesicular glutamate transporter, VGluT1, is increased by corticosterone (F_1,20_ = 60.72, *p* < 0.0001). There was no difference in VGluT1 mRNA expression between KO/CORT and WT/CORT (Fig 6B). These data suggest that chronic corticosterone treatment reduces the efficiency of glutamate reuptake and that neuropsin inactivation partially rescues this defect.

Emerging evidence suggests NMDA receptor dysregulation is implicated in stress induced depressive-like behaviours [38]. To better understand the interaction of neuropsin and corticosterone in regulating NMDA receptors, we measured different subtypes of NMDA receptors in the hippocampus, including 2A (GluN2A) and 2B (GluN2B). KO/CORT mice had significantly lower expression of GluN2B compared to WT/CORT (*p* = 0.006, *t* = 3.44) but no significant difference in GluN2A. The protein expression of NR2B showed a similar pattern to gene expression but there was no significant difference between WT/CORT and KO/CORT. We also detected a lower level of Camk2b gene (*p* = 0.014, *t* = 2.93) and CamKII protein (*p* = 0.036, *t* = 2.41) expression in KO/CORT mice compared to WT/CORT mice but no difference in Camk2a expression (Fig 6B, 6G and 6H). These results suggest that neuropsin inactivation may prevent increased calcium influx seemingly induced by corticosterone injection.

Previous studies show that activation of NMDA receptors induces reactive oxygen species (ROS) production in neurons [39]. Evidence also suggests high levels of plasma corticosterone triggers ROS production leading to oxidative damage in the hippocampus and impairment of memory retention [40]. To test this hypothesis, we conducted a NADPH oxidase-dependent ROS test. In the oil treated groups, our results show no difference in superoxide anion production between WT/oil (n = 13) and KO/oil (n = 15) mice. However, KO/CORT mice (n = 9) have lower superoxide anion in the hippocampus compared to WT/CORT (n = 10) induced by NADPH (*p* = 0.013, *t* = 2.75) (Fig 6C). This suggests that neuropsin inactivation reduces ROS generation resulting from prolonged exposure to a high level of plasma corticosterone.

## Discussion

In this study, we set out to investigate the role of neuropsin in depressive-like behaviour following chronic stress. We observed significant differences in depressive-like behaviour between neuropsin KO and WT mice. However these differences were only evident after animals had experienced a chronic stress regime. Neuropsin is highly expressed in the hippocampus; a region with high neuronal plasticity that has long been considered to be involved in stress, depression, learning and memory. From previous studies, it is unclear whether neuropsin plays a role in learning and memory. One report suggested that neuropsin knockout mice exhibit impairment in the water maze test and that neuropsin is necessary for establishment of LTP, thereby playing a significant role in memory acquisition [20]. However, another study shows no difference in water maze behaviour between KO and WT mice [21]. In our study, intriguingly, we did not detect any difference in learning and memory between WT and KO mice prior to chronic stress. However, after experiencing chronic stress, KO mice performed better in memory tasks than WT mice. Chronic stress leads to disorders such as depression through prolonged elevated glucocorticoids and it is well known that depression may alter the function of learning and memory [17]. From our previous study, we showed that a combination of different stressors leads to depressive-like behaviours in mice [41]. In this study, we used a combination of restraint, foot shock, and sleep deprivation stressors. We confirmed that this array of stressors elicited an increase in plasma corticosterone. We also confirmed that there was no significant difference in plasma corticosterone level between WT and KO groups at each stage of the experiment: before, during and after stress. Our data show that inactivation of neuropsin protects mice from developing depressive-like behaviours and limited the impairment of memory following stress, possibly due to the interaction between neuropsin and corticosterone.

Neuropsin has been shown to be involved in anxiety following acute stress by regulating the dynamics of the EphB2-NMDA-receptor interaction [6]. This suggests there may be an interaction between neuropsin and corticosterone in emotional response. In order to investigate the interaction between neuropsin and corticosterone on depressive-like behaviour and memory, we mimicked one aspect of the hormonal state observed during prolonged stress by injecting mice with a high dose of corticosterone. After chronic corticosterone injection, we observed similar effects to that observed in the chronically stressed animals. Our results suggest that the effects of stress in neuropsin-deficient mice on emotional behaviours and memory performance may be mediated by elevated plasma corticosterone. Additionally, it is also well known that stress or elevated corticosterone can result in excitotoxicity and the modification of hippocampal architecture including decreased dendritic spine density, neurogenesis, and myelination [14, 15, 42, 43]. Our data suggest neuropsin deficient mice are protected against the effects of prolonged elevated corticosterone. This is supported by an assessment of neuronal activity as measured by c-Fos expression. Previous evidence demonstrated elevated plasma corticosterone can be detected more than twelve hours after subcutaneous corticosterone injection [44]. Consequently, we measured c-Fos expression twenty-four hours after the last corticosterone injection in order to assess neuronal activity following our chronic corticosterone treatment. KO mice featured reduced expression of c-Fos in CA1 and CA3, an area where neuropsin is highly expressed in WT after chronic corticosterone treatment. This suggests that inactivation of neuropsin prevents the occurrence of neuronal hyperactivity following chronic corticosterone injection. This may explain why neuropsin KO mice exhibit milder behavioural defects after chronic corticosterone injection than WT. By intra-hippocampal injection of viral vectors, we are able to understand the function of neuropsin more specifically within the hippocampus. In our study, we used a lentiviral vector to knockdown neuropsin on C57BL/6 mice. Additionally we used an AAV9 vector to overexpress neuropsin in the hippocampus of KO mice. Our data support that the development of depressive-like behaviour following chronic corticosterone injection is mediated by the presence of neuropsin.

Results from gene expression and protein quantification of GR reveal KO mice exhibit a reduced response to elevated corticosterone. This can also be confirmed by Sgk1 gene expression. Sgk1 is a direct GR target gene and has been shown to inhibit neurogenesis and be increased in expression in unmedicated depressed patients [33]. A previous study has also shown that Sgk1 up-regulates the expression of NMDA receptor subunit GluN2A and GluN2B [34]. Interestingly, we detected higher levels of gene expression for GluN2B in WT/CORT mice compared to KO/CORT mice, although no significant difference in protein level was observed via western blot. Previous studies have shown GluN2B receptors regulate depressive-like behaviours and mediate excitotoxic neuronal damage [45, 46]. Moreover, WT/CORT mice also exhibited higher levels of CamkII, indicating increased levels of intracellular calcium [47]. WT/CORT mice also have lower expression of EAAT1. This may lead to synaptic glutamate accumulation. Excess glutamate at synapses and an increased influx of calcium ions may trigger downstream responses that increase ROS production [39]. This hypothesis is supported by our ROS experiment. WT/CORT mice display higher level of NADPH dependent ROS in the hippocampus compared to KO/CORT mice. It has been suggested that imbalance between the production of free radicals and the antioxidant capacity of an organism, may contribute to the neuropathology of neurological and psychiatric diseases, including major depression [48]. In our study, repeated corticosterone injection increases ROS production capacity in the hippocampus. This may contribute to the neuronal and behavioural alterations that we have observed. Our data suggests neuropsin inactivation protects against corticosterone induced impairments in hippocampal neuronal architecture and the development of depressive-like behaviours. The lower ROS production capacity of the KO mice chronically injected with corticosterone may help explain the protective effects we observe with this model.

In conclusion, we have identified a novel link between the serine protease neuropsin, chronic stress and depression. We show that neuropsin inactivation has protective effects against depressive-like behaviours and partially rescues spatial memory after stress. These effects could be due to an interaction between elevated corticosterone and neuropsin. Overall, we provide a gene–environment interaction study suggesting that blockade of neuropsin activity reduces the impairment caused by chronic stress or elevated plasma corticosterone. This pathway may consequently be a strong candidate for clinical interventions in the treatment of stress disorders.

## Materials and Methods

### Animals

Experiments were performed on eight-week-old littermate wild-type and neuropsin knockout mice [49] with an equal mixed gender balance. Mice were maintained in specific pathogen-free conditions. They were housed in a 12:12 hour light dark cycle at a temperature of 22°C and a humidity level of 60–70%. Animals had *ad libitum* access to food and water. All the protocols to proceed in this study were reviewed and approved by the Institutional Animal Care and Use Committee at Chang Gung University (Permit Number: CGU14-060).

### Stress-inducing procedure

The stress procedure combined restraint stress, foot shock, and sleep deprivation. Animals were subjected to 3 hours restraint stress on the first day. The following day they were placed in a plexi glass foot shock chamber and given two inescapable 1 mA foot shocks lasting 5 seconds within a period of 3 minutes. The next day, mice were subjected to sleep deprivation by being placed in a water tank containing multiple and visible platforms (4.5 cm in height and diameter) surrounded by water for 12 hours [50]. This battery of stress inducing paradigms was repeated for 28 days.

### Behaviour tests

Novelty suppressed feeding: Food pellets were removed from the home cage 24 hours before testing. Mice were placed for 7 min in a novel cage with food pellets placed in the 4 corners and a bright light positioned above to illuminate the whole testing chamber. All activity was tracked by a video tracker. Both the latency to initiate eating and the time spent eating were measured.

Forced swim test: Mice were put into a water tank (diameter = 20 cm), containing 15 cm of water for 6 min and their immobility time was measured.

Morris water maze: The water maze has four starting positions: north, south, east, and west. Before beginning the test we chose the order of the starting directions for consistency: north, east, south and then west. For training, mice were placed in the maze and allowed to swim/search for the hidden platform for a maximum of 90 seconds. The time taken to reach the platform was recorded. If the mice did not find the platform within 90 seconds they were guided to the platform. This procedure was repeated three times for each starting direction over three days (12 trials in total). We then performed the memory test 14 days after training was complete, with the platform removed. We recorded the time each mouse spent in the correct quadrant (target zone) where the training platform was previously located. We also recorded how many times the mice crossed the phantom platform location.

Sucrose preference: For a habituation period of three days, mice were presented with two drinking bottles; 1% sucrose solution and drinking water. The position of the two bottles was switched every day to reduce side bias. Following habituation, mice were not given water for 6 hours prior to testing. We measured the net weight of both water (W0) and sucrose (S0) before the test and 24 hours later (W1, S1) when the test was completed. The preference (sucrose consumption %) was calculated by the following formula:

Sucrose consumption(%)=(S0−S1)/ [(S0−S1)+(W0−W1)]*100.

### Corticosterone assay

Blood samples were collected to measure basal corticosterone level and the level after 3 hrs of restraint stress, 30 min after footshock, after 6 hrs of sleep deprivation and 24 hrs after all stressors via facial vein puncture. Plasma was separated from whole blood by centrifugation (3000g, 4°C, 10 min) and stored at -80°C until used. Plasma was diluted 1:30 in buffer and measured using the Corticosterone EIA kit (Enzo Life Sciences) according to the manufacturer’s instructions.

### Immunohistochemistry

Brains were harvested and fixed in 4% PFA (pH7.4) for a day. The brains were then dehydrated for 24 hrs in 25% sucrose solution. All sections for KI67, DCX, c-Fos and CNPase staining were cut to a thickness of 30 μm on a sliding microtome. Sections were mounted on superfrost slides and dried overnight. Subsequently, slides were incubated in 0.01 mol/L citric buffer for 20 min at 90°C, 3% H_2_O_2_ for 10 min, rinsed in PBS, and incubated overnight at room temperature in rabbit anti-KI67 antibody (1:4000, Vector Lab), goat anti-DCX antibody (1:250, Santa Cruz), rabbit anti-c-Fos (1:1000, Santa Cruz), or rabbit anti-CNPase (1:1000, Abcam). The next day, a standard IgG ABC kit (Vector Lab) procedure was used and the slides reacted for 5–10 min with a Sigma DAB tablet. Sections were then counterstained with cresyl violet and cover-slipped with DPX.

For BrdU staining, following a 3% H_2_O_2_ incubation for 10 min, slides were subsequently incubated in 2M HCL for 30 min at 37°C. Rat anti-BrdU antibody (1:250, Accurate) was applied overnight. The next day the ABC kit procedure was followed and the slides were reacted with a Sigma DAB tablet. Hippocampus cells were counted bilaterally on every eighth section through the entire rostrocaudal extent of the granule cell layer.

### Quantification

All slides were randomized and coded before quantitative analysis. Slides (half brain) were examined under a 20× objective. DCX, KI67 and BrdU labelled cells were counted on every eighth section through the entire rostrocaudal extent of the granule cell layer (6 sections per animal). The number of cells counted was then multiplied by sixteen to obtain an estimate of the total number of DCX, KI67 and BrdU positive cell in the dentate gyrus. For c-Fos labelled cells, we counted cells on every eighth section through the entire rostrocaudal extent of the dentate gyrus, CA1 and CA3 areas (6 sections per animal). The raw data was then modified as detailed above.

### Golgi staining

Brains were harvested and stained using the superGolgi Kit (Bioenno Tech) in accordance with the manufacturer's instructions. Brains were impregnated with solution for 9 days. This was followed by 2 days of dehydration in sucrose. Brains were subsequently sectioned coronally at a thickness of 150 μm using a microtome. We calculated the spine density of the secondary branches of pyramidal neuron apical dendrites in CA3. We selected a total of 18 neurons per each animal. Three different neurons were randomly selected for measuring per brain slice in both WT and KO mice (n = 4). A total of six brain slices were analysed per animal.

### Viral vector preparation and intra-hippocampal injections

For the neuropsin overexpression experiment and control, DNA fragments that encoded KLK8 or EGFP were created by PCR and subcloned into the NotI site of a AAV9 virus construct. Recombinant AAV9 vectors were produced by a standard triple-plasmid transfection method and purified by two rounds of CsCl centrifugation. The physical vector titres of AAVs were quantified by measuring the number of packaged vector genomes using a real-time PCR method. For the neuropsin knockdown experiment and control, a klk8 short hairpin RNA (shRNA) lentiviral vector and scrambled sequence control vector were obtained from the National RNAi core facility (Institute of Molecular Biology, Academia Sinica, Taiwan). The target sequence for Klk8 shRNA was: CCTCAACTGTGCGGAAGTGAA.

For the viral vector injections, surgery was performed under anaesthesia (a Zoletil-Rompun mixture). Mice received bilateral injections of viral vectors into hippocampus in a volume of 1.5 μl (5x10^13^ VG/ml for AAV9 viral vector, 5.6x10^6^ RIU/ml for lentiviral vector) under stereotaxic guidance. There were four injection sites in total (For dorsal hippocampus, AP– 2.2mm, ML ± 2.0 mm from bregma and DV—1.8 mm from dura; for ventral hippocampus, AP—3.0mm, ML ± 3.0 and DV—3.3 mm). (S4A Fig)

### mRNA quantification

Hippocampal tissue was collected from WT and KO mice. mRNA was extracted using a RNeasy Lipid Tissue Kit (Qiagen). cDNA was then made using SuperScript III reverse transcriptase (Invitrogen). Experiments were performed in duplicate. Gene expression levels were then calculated by the ^ΔΔCt^ method and normalized against a Gapdh control. Primers used in this study are in the Table 1.

**Table 1:** Primers used for quantification of mRNA expression.

	Forward	Reverse
NP (*Klk8*)	CGTGGATCCTTCTGCTTCTG	CCCCCACAGATCAGTCTCTC
GR (*Nr3c1*)	ACGCCGACTTGTTTATCTGG	GAAAAGGACGCCAGACTCC
*Sgk1*	CTGCTCGAAGCACCCTTACC	TCCTGAGGATGGGACATTTTCA
*Fkbp5*	AGCACACATCCCGTGTTCTA	TGCTGGGTTCTCTCCATTGT
*GluN2A*	ACGTGACAGAACGCGAACTT	TCAGTGCGGTTCATCAATAACG
*GluN2B*	GCCATGAACGAGACTGACCC	GCTTCCTGGTCCGTGTCATC
*VGluT1*	GGTGGAGGGGGTCACATAC	AGATCCCGAAGCTGCCATAGA
EAAT1	ACCAAAAGCAACGGAGAAGAG	GGCATTCCGAAACAGGTAACTC
*CamK2a*	TGCCTGGTGTTGCTAACCC	CCATTAACTGAACGCTGGAACT
*CamK2b*	GCACGTCATTGGCGAGGAT	ACGGGTCTCTTCGGACTGG
*Cnp*	ACCATTGTCCCCCTATACCC	TATGTATGGGCAAAGCCACA
*Mog*	AGCTGCTTCCTCTCCCTTCTC	ACTAAAGCCCGGATGGGATAC
*Gapdh*	TGACGTGCCGCCTGGAGAAAC	CCGGCATCGAAGGTGGAAGAG

### Western blot analysis

Protein extracts were obtained by lysing tissue in 1% SDS. Samples were homogenized and heated at 100°C for 10 min. Proteins were then separated by sodium dodecyl sulphate–polyacrylamide gel electrophoresis and electro-transferred onto nitrocellulose membranes. Blots were incubated with primary antibody (GR 1:1000, Santa Cruz; SGK1 1:1000, BOSTER; Fkbp5 1:3000, Santa Cruz; NR2b 1:1000, Millipore; CamKII 1:1000, Santa Cruz; EAATI 1:1000, Alpha Diagnostic) placed in TBST and 5% non-fat milk overnight at 4°C. Subsequently, blots were washed and probed with the respective horseradish peroxidase secondary antibody for 1 h at room temperature. The immunoreactive bands were visualized using ECL detection reagent (GE Healthcare, RPN2106). Assessment of the band intensities were performed using the ChemiDoc MP from Bio-Rad.

### Superoxide anion analysis

Superoxide anion production in the hippocampus tissues was measured by modified lucigenin-enhanced chemiluminescence. This method to test superoxide anion production level in tissue is based on the nitro blue tetrazolium (NBT) reduction method [51]. Hippocampi were isolated, chopped and then placed in a white plate containing Krebs buffer. Lucigenin (25 μM) was added to test the basal level of superoxide anion production. NADPH (500 μM) was then added to stimulate NADPH-oxidase dependent ROS production. The magnitude of superoxide anion production was normalized for the dry weight of the chopped hippocampus.

### Statistical analysis

The mean ± SEM was determined for each group. Statistical analyses were performed using Graphpad Prism software. Data were analyzed via an analysis of variance (ANOVA) or *t*-test as appropriate. Fisher's LSD method was performed when applicable. Differences were considered significant when *p* was less than 0.05.

## Supporting Information

S1 Fig No significant difference in open field activity between WT and neuropsin KO mice. (A) No significant basal activity difference between WT and KO mice. However, chronic stress significantly decreased open field activity (B) No significant difference in activity between WT and KO following oil injection, however, corticosterone injection greatly decreased activity in the open field. Values represent mean ± SEM. * *p* < 0.05.
(TIFF)

S2 Fig Neuropsin deficient mice exhibit no significant difference in plasma corticosterone level compared to WT mice before, during and after stress. Plasma corticosterone on WT and KO mice (n = 8) before stress, after 3hrs of restraint stress, 30 min after footshock, after 6 hrs of sleep deprivation and 24 hrs after all stressors had ceased. Stress strongly elevated plasma corticosterone level but there was no significant effect of neuropsin on these levels. Values represent mean ± SEM.
(TIF)

S3 Fig Viral vectors mediated expression of neuropsin in the hippocampus with chronic corticosterone treatment alters depressive-like behaviour and spatial memory. Fourteen days after intrahippocampal viral vector injections, all the mice were given daily corticosterone injections. Two weeks later, all mice were subjected to behaviour tests. (A) Hippocampal overexpression of neuropsin in KO mice (NP/OE, n = 8) increases depressive-like behaviour as measured by the novelty suppressed feeding test and forced swim test, although there is no significant difference in the sucrose preference test compared to neuropsin KO mice (NP/KO, n = 8) (B) No significant difference in the water maze learning curves between NP/OE and NP/KO mice. Two weeks after water maze experiment, NP/OE mice exhibited fewer phantom platform crosses in a memory test. (C) Knockdown hippocampal neuropsin (n = 9) attenuated the development of depressive-like behaviour in novelty suppressed feeding and forced swim tests compared to control mice (n = 9). (D) No significant difference in the water maze learning curves between control and knockdown mice was observed, however, there was a significant difference in the number of phantom platform crosses during the memory test. Values represent mean ± SEM. * *p* < 0.05, ** *p* < 0.01, *** *p* < 0.001.
(TIF)

S4 Fig Viral vectors mediate the expression of hippocampal neurogenesis. (A) The injected dye shows the positioning of the intrahippocampal injection. (B) KO mice injected with neuropsin overexpression viral vector (NP/OE) in the hippocampus feature significantly increased hippocampal neuropsin mRNA compared to WT (NP/WT) and KO (NP/KO) mice (n = 5 in each group). (B((Mice injected with neuropsin shRNA lentiviral vector feature significantly decreased neuropsin expression in the hippocampus (n = 6 in each group). Values represent mean ± SEM. * *p* < 0.05, *** *p* < 0.001.
(TIFF)
