# Supplementary figures and images for: Neuropsin Inactivation Has Protective Effects against Depressive-Like Behaviours and Memory Impairment Induced by Chronic Stress
Source: PLoS Genet. 2016 Oct 4;12(10):e1006356. doi: 10.1371/journal.pgen.1006356 (PMC5049781; doi:10.1371/journal.pgen.1006356)

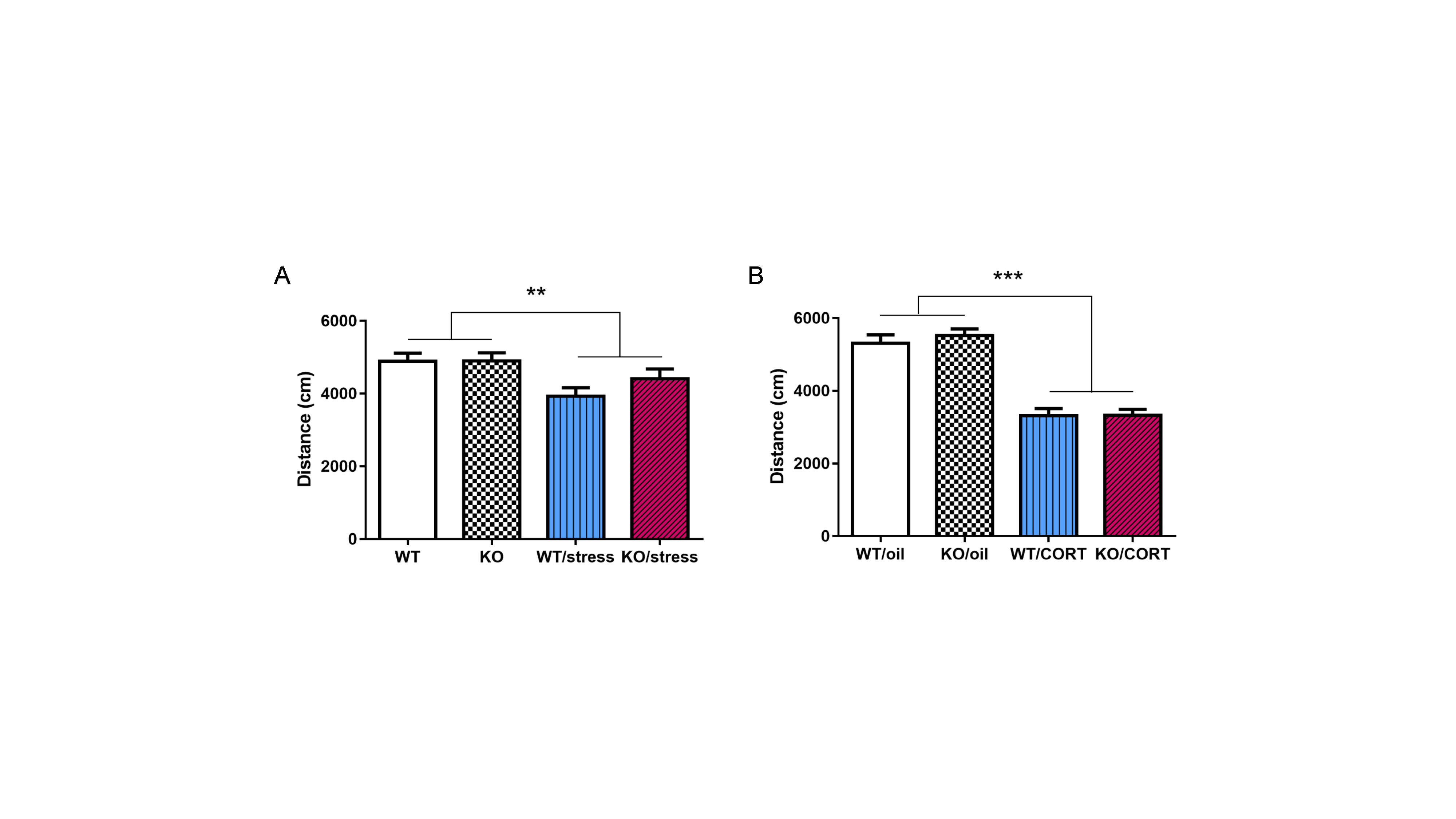

Supplement: S1 Fig — (A) No significant basal activity difference between WT and KO mice. However, chronic stress significantly decreased open field activity (B) No significant difference in activity between WT and KO following oil injection, however, corticosterone injection greatly decreased activity in the open field. Values represent mean ± SEM. * p < 0.05. (TIFF) [file pgen.1006356.s001.tiff]

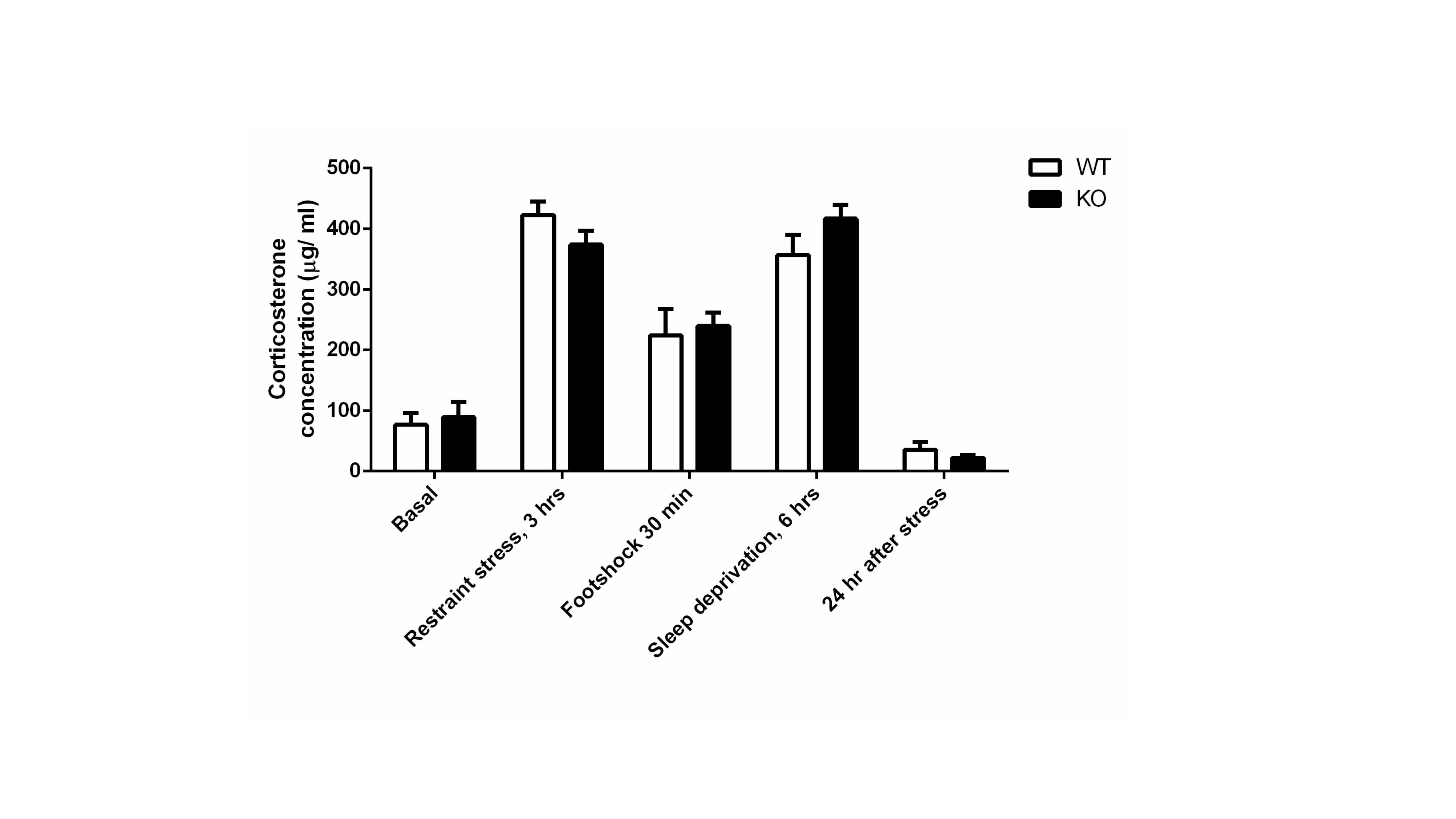

Supplement: S2 Fig — Plasma corticosterone on WT and KO mice (n = 8) before stress, after 3hrs of restraint stress, 30 min after footshock, after 6 hrs of sleep deprivation and 24 hrs after all stressors had ceased. Stress strongly elevated plasma corticosterone level but there was no significant effect of neuropsin on these levels. Values represent mean ± SEM. (TIF) [file pgen.1006356.s002.tif]

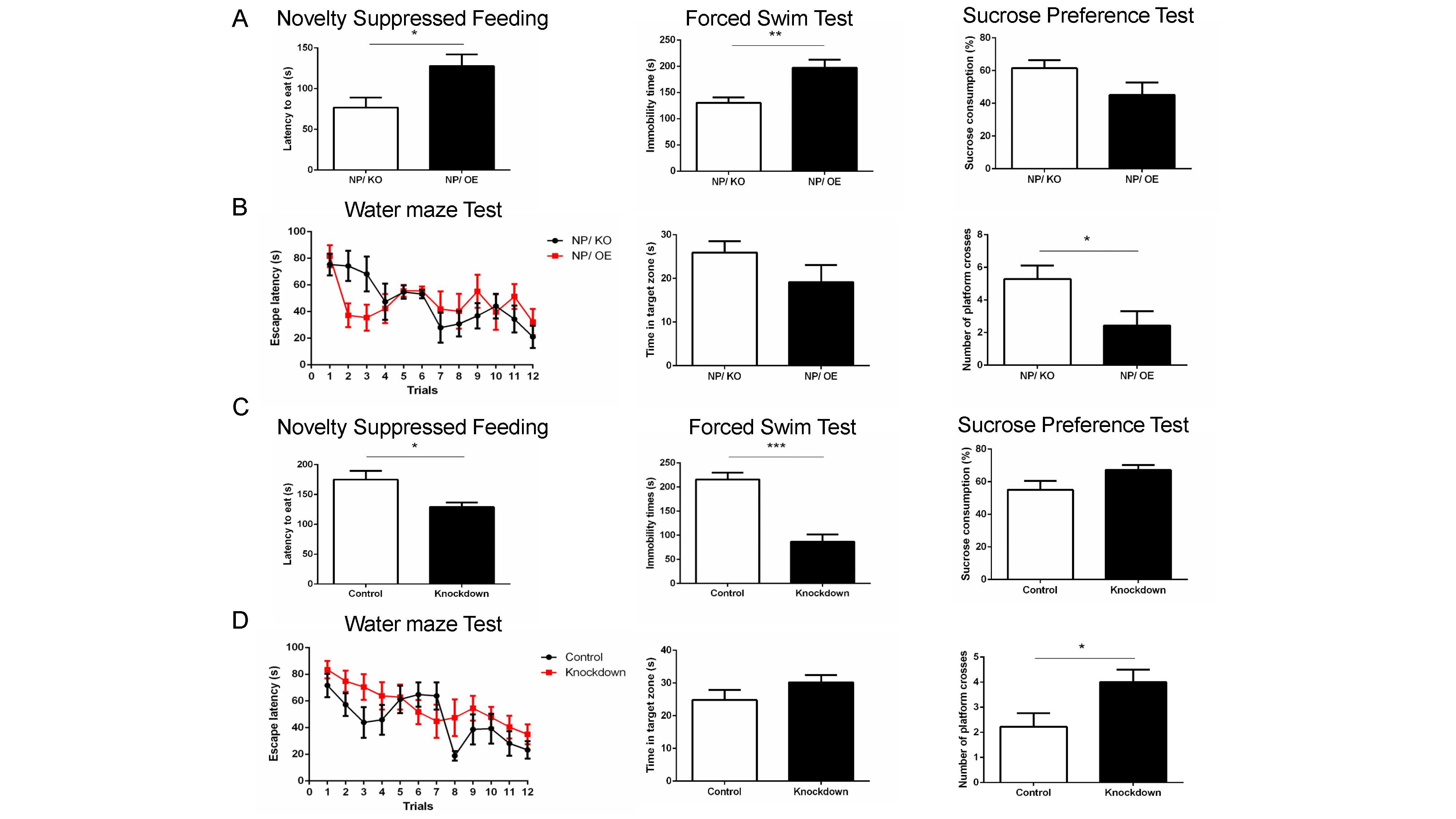

Supplement: S3 Fig — Fourteen days after intrahippocampal viral vector injections, all the mice were given daily corticosterone injections. Two weeks later, all mice were subjected to behaviour tests. (A) Hippocampal overexpression of neuropsin in KO mice (NP/OE, n = 8) increases depressive-like behaviour as measured by the novelty suppressed feeding test and forced swim test, although there is no significant difference in the sucrose preference test compared to neuropsin KO mice (NP/KO, n = 8) (B) No significant difference in the water maze learning curves between NP/OE and NP/KO mice. Two weeks after water maze experiment, NP/OE mice exhibited fewer phantom platform crosses in a memory test. (C) Knockdown hippocampal neuropsin (n = 9) attenuated the development of depressive-like behaviour in novelty suppressed feeding and forced swim tests compared to control mice (n = 9). (D) No significant difference in the water maze learning curves between control and knockdown mice was observed, however, there was a significant difference in the number of phantom platform crosses during the memory test. Values represent mean ± SEM. * p < 0.05, ** p < 0.01, *** p < 0.001. (TIF) [file pgen.1006356.s003.tif]

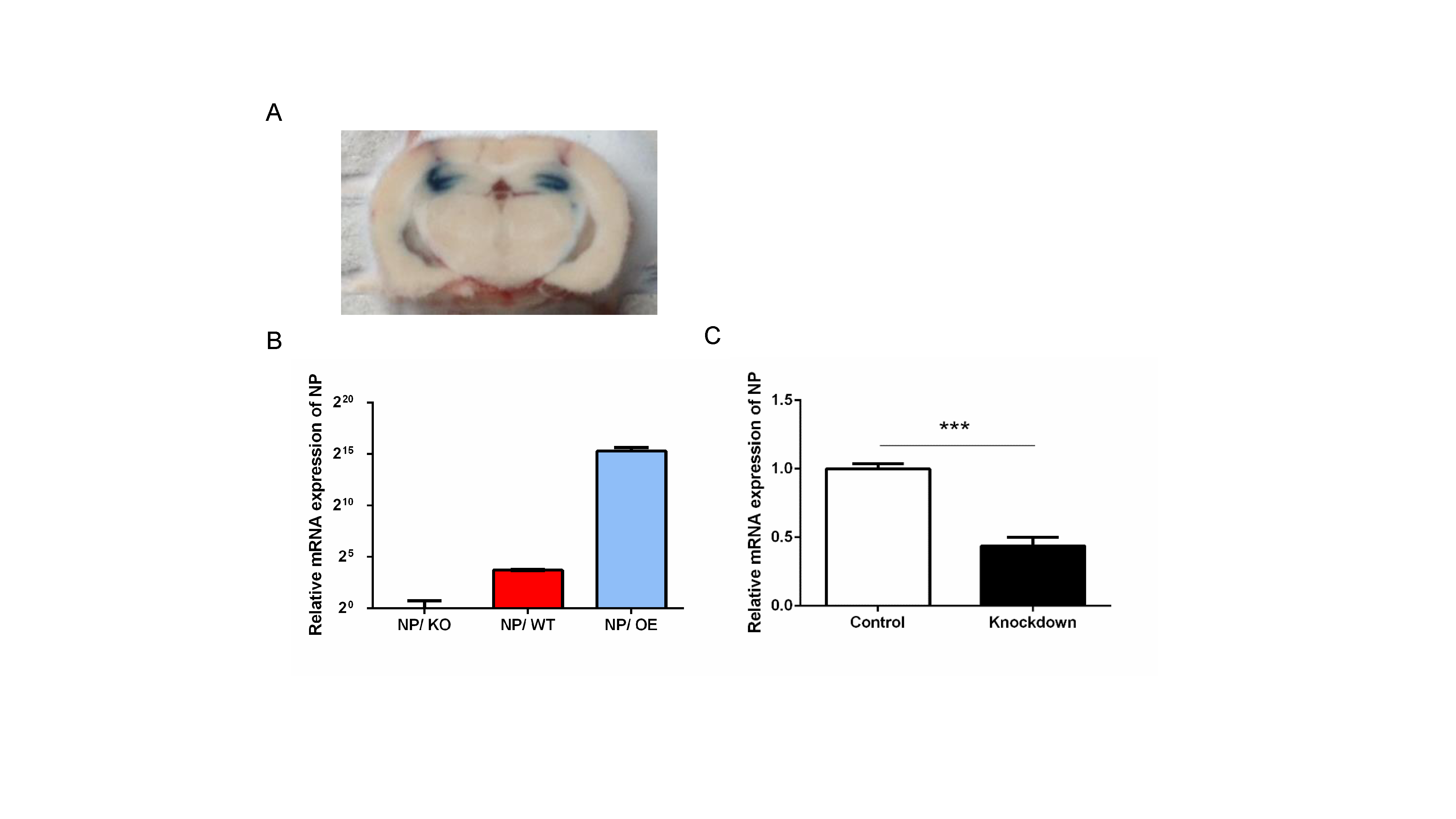

Supplement: S4 Fig — (A) The injected dye shows the positioning of the intrahippocampal injection. (B) KO mice injected with neuropsin overexpression viral vector (NP/OE) in the hippocampus feature significantly increased hippocampal neuropsin mRNA compared to WT (NP/WT) and KO (NP/KO) mice (n = 5 in each group). (B((Mice injected with neuropsin shRNA lentiviral vector feature significantly decreased neuropsin expression in the hippocampus (n = 6 in each group). Values represent mean ± SEM. * p < 0.05, *** p < 0.001. (TIFF) [file pgen.1006356.s004.tiff]
